# Nova food classification system: a contribution from Brazilian epidemiology

**DOI:** 10.1590/1980-549720250027

**Published:** 2025-06-02

**Authors:** Maria Laura da Costa Louzada, Kamila Tiemann Gabe

**Affiliations:** I Universidade de São Paulo, Center for Epidemiological Research in Nutrition and Health – São Paulo (SP), Brazil.

**Keywords:** Nutritional epidemiology, Eating, Processed food, Nutritional policy, Food guides

## Abstract

This essay presents the Nova food classification, a conceptual innovation of Brazilian epidemiology, describing its genesis, the scientific evidence derived from its application, and its implications for public health. Created in 2010, Nova classifies foods into four groups based on the degree of processing: unprocessed or minimally processed foods, processed culinary ingredients, processed foods, and ultra-processed foods. Since its development, several epidemiological studies have demonstrated the negative impacts of high consumption of ultra-processed foods on health, such as associations with various non-communicable diseases, including obesity, diabetes, cardiovascular diseases and mental health outcomes. In Brazil, the consumption of these foods has increased significantly in recent decades, with the caloric share of ultra-processed foods rising from 12.6 to 18.4% between 2002–2003 and 2017–2018, with this increase being more pronounced among more vulnerable socioeconomic groups. Nova underpins the recommendations of the Brazilian Dietary Guidelines and has played a crucial role in informing public policies, such as the update of the National basic food basket and the guidelines of the National School Feeding Program, which aim to limit the access to ultra-processed foods. Finally, the essay addresses the political and scientific challenges, including the need for more experimental studies to strengthen the evidence and the potential of fiscal and marketing regulation strategies that take into account the impact of food processing on health.

## INTRODUCTION

In the late 1980s, Household Budget Surveys began to reveal notable changes in the food purchasing patterns of Brazilian families. From 1987 to 2003, there was a significant drop in the purchase of some foods such as rice, beans, flour, eggs and meat, as well as culinary ingredients, such as table salt, table sugar and vegetable oils. At the same time, there was an increase in the purchase of industrialized cakes, snacks and cookies, sausages, ready-to-eat meals and sweetened beverages^1^.

At that time, industrial processing was practically ignored in studies on food consumption, and the initial conclusions seemed to lead to a paradox: despite the drop in the purchase of salt, oils and sugar — the reduction of which was a central recommendation to combat obesity and chronic non-communicable diseases (NCDs) — these health problems continued to increase. Investigations motivated by this discrepancy revealed that a radical transition was underway in the dietary patterns of the Brazilian population.

The decline in the purchase of these foods, contrary to what would be expected, was not accompanied by a change in the nutritional profile of the diet, i.e., a reduction in the consumption of sodium, free sugar and fats, but rather indicated a reduction in the consumption of traditional foods and their culinary preparations, such as rice and beans (whose preparation requires culinary ingredients), and the concomitant increase in the consumption of industrialized products.

According to the observation that a new paradigm would be needed to understand this transition, the team at the Center for Epidemiological Research in Nutrition and Health at the University of São Paulo (NUPENS/USP) began in 2009 a revolutionary line of research based on the hypothesis that industrial processing was a key factor in the relationship between food and health^2^.

## NOVA FOOD CLASSIFICATION SYSTEM

In 2010^3^, the Nova food classification was proposed, which, after updates over the years, divides all foods according to industrial processing characteristics into four groups: fresh or minimally processed foods, processed culinary ingredients, processed foods and ultra-processed foods (Chart 1).

**Chart 1.** Definition and examples of the four groups of the New Food Classification.

**Group 1) Fresh or minimally processed foods.**
Fresh foods are edible parts of plants (fruits, seeds, leaves, stems, roots, tubers) or animals (muscles, viscera, eggs, milk), as well as fungi, algae and water, after separation from nature. Minimally processed foods are foods altered by minimal industrial processes, such as removal of inedible or unwanted parts, drying, milling, shredding, roasting, fractionating, toasting, pasteurization, refrigeration, freezing, non-alcoholic fermentation and other methods that do not add salt, sugar, oils or fats or other ingredients to the original food. These processes aim to extend the shelf life of these foods, allowing their storage for prolonged use and, often, to facilitate or diversify their preparation. Additives are generally not necessary and are found only exceptionally in minimally processed foods. Examples: Fresh, juiced, chilled, frozen or dried fruits and leafy and root vegetables; grains such as brown, parboiled or white rice, corn on the cob or kernels, wheat grains; legumes such as beans, lentils and chickpeas; raw or roasted nuts and oilseeds (no added salt or sugar); roots and tubers such as potatoes, sweet potatoes and cassava; fungi such as fresh or dried mushrooms; herbs and spices such as thyme, oregano, mint, pepper, cloves and cinnamon, whole or powdered, fresh or dried; corn, wheat, oat or cassava groats, flakes or flour; meat, poultry, fish and seafood, whole or cut, fresh, chilled or frozen; eggs; fresh or pasteurized milk; fresh or pasteurized natural yoghurt; fresh or pasteurized fruit or vegetable juices (no added sugar, sweeteners or flavorings); tea, coffee and drinking water. In addition, foods composed of two or more items from this group, such as mixed dried fruits, granola made from cereals, nuts and dried fruits without added sugar, honey or oil; pasta, couscous and polenta made with flours, flakes or semolina and water; and foods with added vitamins and minerals, usually to replace nutrients lost during processing, such as wheat or corn flour fortified with iron and folic acid.
**Group 2) Processed culinary ingredients.**
Substances obtained directly from group 1 foods or from nature by processes such as centrifugation, dehydration, extraction, mining, pressing and refining. These processes help in the creation of products used in seasoning and cooking group 1 foods and their use in culinary preparations. Additives are generally not necessary and are found only exceptionally in processed culinary ingredients. Examples: Vegetable oils extracted from seeds, nuts or fruits (notably olives); sugar and molasses obtained from sugar cane or beets; honey extracted from combs and syrup from maple trees; vinegar; starches extracted from corn and other plants; salt extracted from mines or sea water; butter and lard obtained from milk and pork. In addition, products composed of two items from group 2, such as salted butter, and items from group 2 with added vitamins or minerals, such as iodized salt.
**Group 3) Processed foods.**
Industrially manufactured food products made by adding at least one ingredient from group 2 (such as salt, sugar, oil or fat) to foods from group 1, through preservation methods such as canning and bottling and, in the case of bread and cheese, using non-alcoholic fermentation and boiling or baking. The processes and ingredients aim to increase the shelf life of group 1 foods and modify or enhance their sensory qualities. Processed foods often contain additives that extend the shelf life of the product, protect original properties or prevent the proliferation of microorganisms (such as preservatives and antioxidants), but not additives with cosmetic functions (present in ultra-processed foods). Examples: Fruits in syrup; vegetables and pulses canned or bottled in brine; salted or sweetened nuts and seeds; dried or canned fish. Breads, cheeses, pastries, cakes, biscuits; cured meats; and ready-to-heat products, such as hamburgers, pies and pre-prepared pasta and pizza dishes, when these products are made exclusively from foods in group 1 and salt, oil, sugar or other ingredients in Nova group 2 and do not contain classes of additives with a cosmetic function.
**Group 4) Ultra-processed foods.**
Industrially manufactured food products composed of several ingredients (formulations), including oils, fats, sugar and salt (usually in combination and in larger quantities than in processed foods) and food substances of rare or non-existent culinary use (such as hydrogenated oils, modified starches, high fructose corn syrup and protein isolates). Group 1 foods are absent or represent a small proportion of the ingredients in the formulation. The processes that allow the manufacture of ultra-processed foods include industrial techniques such as extrusion, molding and pre-frying; application of additives, including those whose function is to make the product palatable or hyperpalatable, such as colors, non-sugar sweeteners, flavors and emulsifiers; and sophisticated packaging, usually with synthetic materials. The processes and ingredients here are designed to create highly profitable (low-cost ingredients, long shelf life, emphatic branding) and convenient (ready to eat or drink) alternatives to all other Nova food groups. Ultra-processed foods are operationally distinguishable from processed foods by the presence of food substances of rare or nonexistent culinary use (varieties of sugars, such as high fructose corn syrup, fructose, invert sugar, fruit juice concentrates, dextrose, maltodextrin and lactose; modified starches; modified oils, such as hydrogenated or interesterified oils; and protein sources, such as gluten, soy protein isolate, casein, whey, hydrolyzed proteins and mechanically separated meat) or additives with cosmetic functions (flavor enhancers, aromas, colorants, emulsifiers, sweeteners, emulsifying salts, thickeners and bulking agents, carbonation, foaming, gelling, icing, anti-foaming agents). Examples: Reconstituted fruit juices and fruit drinks; carbonated soft drinks; dairy drinks and energy drinks; flavored yogurt; confectionery; margarines; sausages, hot dogs, chicken and fish nuggets, reconstituted meat products; plant-based meat substitutes; extruded breakfast cereals; instant soups, noodles and powdered desserts; infant formulas and follow-on milks; health and weight loss products, such as meal replacement shakes and powders. Breads, pastries, cakes, cookies; cured meats; sweet or savory snacks; and ready-to-heat products, such as pre-prepared hamburgers, pies, pasta dishes and pizza, when these products are composed of food substances of rare or nonexistent culinary use and/or contain classes of additives with a cosmetic purpose.

Source: Adapted from Martinez-Steele et al.^4^.

Ultra-processed foods are defined as ready-to-eat industrial formulations, made with several stages of industrial processing and parts of food components (such as fats, sugars, starch, isolated proteins), with little or no presence of the original food matrix and often with many cosmetic additives that make them more attractive. A practical way to identify these foods is by analyzing the list of ingredients, looking for one of the following markers:

- cosmetic food additives, which modify the sensory characteristics of the product, such as flavoring, colorant, sweetener, thickener, flavor enhancer;
- substances rarely used in cooking, such as fructose, high-fructose corn syrup, fruit juice concentrates, invert sugar, maltodextrin, dextrose, lactose, hydrogenated oils, soy protein isolate, casein and mechanically separated meat.

Martinez-Steele et al.^4^ recently proposed the best practices for applying the Nova classification, aiming to improve the accuracy of categorization, especially when detailed information about the origin, method of preparation and ingredients present in the food is missing.

## ULTRA-PROCESSED FOODS AND HEALTH: SCIENTIFIC EVIDENCE

The first evidence of the negative impact of replacing fresh or minimally processed foods with ultra-processed foods on health was produced on the basis of data from Brazilian surveys of individual food consumption. The studies showed cross-sectional associations between the high participation of ultra-processed foods in the diet, the deterioration of the nutritional quality of the diet (both in relation to macronutrients and micronutrients) and the greater chance of obesity^5,6,7^. These articles were the basis of the evidence in the Dietary Guidelines for the Brazilian Population, published by the Ministry of Health in 2014^8-9^, with the central recommendation being to reduce the consumption of ultra-processed foods and strengthen the traditional Brazilian dietary pattern based on fresh or minimally processed foods.

As a result, the Nova system started being applied to data from several cohort studies around the world, many with large and long-term samples, which were subsequently reanalyzed in light of the new classification. Several studies were published by independent research groups showing the negative impact of ultra-processed foods on health. Recently, an umbrella review summarized these studies and demonstrated that high consumption of ultra-processed foods was associated with an increased risk of more than 30 negative health outcomes, including diabetes, cardiovascular disease, and even depression^10^. Despite the limitations of observational studies, such as potential residual confounding bias and measurement errors, the association with increased risk for several NCDs was consistently observed in many large, mostly high-quality cohort studies, reinforcing the plausibility of a causal relationship.

Complementary evidence comes from randomized controlled trials (RCTs). Hall et al.^11^ compared the effects of offering an ultra-processed diet (~80% ultra-processed foods) and an unprocessed diet (without ultra-processed foods). Participants were free to eat, but the menus offered were the same in terms of calories, carbohydrates, sugar, fat, sodium and fiber. The results showed that when exposed to ultra-processed diets, participants consumed, on average, 508 kcal more per day than when exposed to diets without ultra-processed foods. After two weeks, the participants gained 0.9 kg of weight consuming the ultra-processed diet and lost the same amount of weight consuming the non-ultra-processed diet^11^. Similar results were observed in a Japanese RCT in which participants who followed an ultra-processed diet for one week gained, on average, 2.2 kg of weight and 0.7 kg of fat mass. In contrast, those who consumed the non-ultra-processed diet gained 1.1 kg of weight but lost 0.4 kg of fat mass^12^.

Furthermore, several studies have begun to raise hypotheses that the mechanisms linking ultra-processed foods to diseases go far beyond their nutrient profile. Studies show that ultra-processed foods, because of their convenience and hyperpalatability, replace healthy fresh or minimally processed foods, such as legumes, vegetables and fruits. The destruction of natural elements in the food matrix by processing techniques compromises the consumption of bioactive compounds that are relevant to cardiovascular health. Ultra-processed foods increase contact with toxic xenobiotics, such as acrylamide and acrolein (newly formed in processing using carbohydrates) and bisphenol A (released from plastic packaging), and the ingestion of food additives, such as colorants and flavorings. The latter, despite being regulated by law, have increasingly proven to be harmful, particularly in the long term. Finally, ultra-processed foods have a low satiety level, can induce behaviors related to addiction and thereby lead to excessive calorie consumption^13,14^.

## TEMPORAL TREND OF CONSUMPTION OF ULTRA-PROCESSED FOODS IN BRAZIL

In Brazil, there has been a trend towards replacing fresh or minimally processed foods and culinary ingredients with ultra-processed foods. A study using food purchase data from the Household Budget Survey revealed an increase in the share of these foods in total calories purchased in the household from 12.6 to 18.4% between 2002–2003 and 2017–2018. In metropolitan areas, this trend has been observed since the 1980s, with an increase from 10 to 24% of total energy purchased in households between 1987–1988 and 2017–2018. The largest growth in the share of ultra-processed foods was observed between 1995 and 2003, followed by a recent slowdown, attributed mainly to the reduction in soft drink purchases between 2009 and 2018^15^.

In another study, the fraction of the increase in the prevalence of obesity attributable to increased consumption of ultra-processed foods in Brazil between 2002 and 2009 was quantified. The comparison of two national surveys, with data on the household purchase of ultra-processed foods and obesity, together with data on the estimated risk associated with the consumption of these foods, showed that the increase in the consumption of ultra-processed foods in the seven-year period was responsible for more than a quarter (28.6%) of the increase in the prevalence of obesity in the same period^16^.

**Figure 1.**
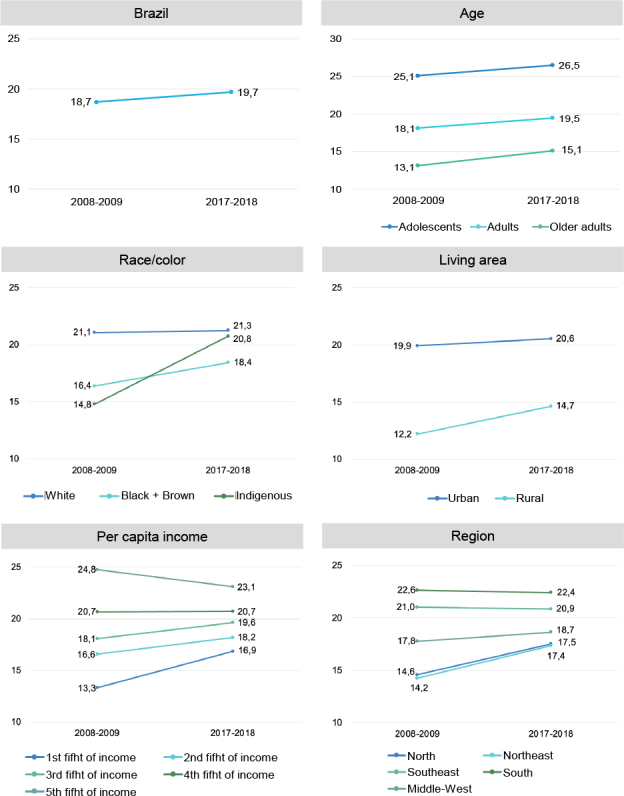
Temporal trend of ultra-processed food consumption (% of total energy) in Brazil according to sociodemographic and economic characteristics. Household Budget Surveys 2008-2009 and 2017-2018.

Source: Adapted from Louzada^17^.

Since 2008, there was also a national survey that assessed participants’ individual consumption inside and outside the. These data revealed that between 2009 and 2017, there was a trend towards an increase in the share of ultra-processed foods in the diet, reaching almost 20% of calories consumed and varying significantly between sociodemographic strata (Figure 1). For example, white people had a higher consumption of ultra-processed foods throughout the period, however, the consumption of ultra-processed foods increased significantly among black and indigenous people, but not among white people. Regarding the area of ​​residence, residents of urban areas have higher consumption, and although the increase was observed in both the urban and rural strata, it was more intense among residents of rural areas^18^.

It was also seen that in Brazil, there is still a direct relationship between income and consumption of ultra-processed foods, with consumption of these foods being higher among wealthier people. However, consumption of ultra-processed foods increased significantly in the three lowest family income groups, stabilized in the upper middle income group and decreased significantly in the highest income group^18^. Therefore, over these 10 years, there was a standardization of national consumption at a higher level, further harming the most vulnerable socioeconomic strata.

## DETERMINANTS OF CONSUMPTION OF ULTRA-PROCESSED FOODS

In Brazil, the rise in the consumption of ultra-processed foods is a relatively recent phenomenon, dating back to the 1980s, when neoliberal economic policies opened the Global South to the entry of transnational ultra-processed food companies. The globalization of the food system has begun to transform local environments^19^.

Among the main drivers of the increase in the consumption of ultra-processed foods is the increase in physical and financial access to these foods. In 1995, ultra-processed foods were the most expensive food group. Since the early 2000s, the price of these foods has undergone successive reductions, narrowing the gap between them and fresh or minimally processed foods^20^. More recent data show that the crisis resulting from the COVID-19 pandemic accelerated this process, and this price reversal is already happening^21^.

Supermarkets have gradually replaced more traditional shopping venues, such as street markets and greengrocers, by offering a wide variety of ultra-processed foods at lower prices than those found elsewhere.^22^ In addition, advertising aimed at low-income communities has also helped accelerate the penetration of these products into the lower socioeconomic strata of the population. Two examples are the door-to-door sales of ultra-processed foods in peripheral communities and the Nestlé ship selling its products in Amazonian communities^23^.

Another crucial factor that favors the increased consumption of ultra-processed foods is the political activity of large corporations in the sector and conflicts of interest. Transnational ultra-processed food industries have vast resources to develop corporate political activities aimed at defending their interests, which directly impacts the formulation and implementation of public health policies. These strategies include direct lobbying of decision-makers, the use of legal instruments to block regulations, communication campaigns aimed at influencing public opinion, and the construction of narratives that highlight the role of the sector in the economy. In addition, actions to strengthen ties with health professionals and researchers are common, seeking to lend credibility to the industry’s positions^24^.

On the other hand, there are opposing forces. They help to explain the fact that the magnitude of consumption observed in Brazil is lower than that observed in other countries, such as the United States, where the consumption of these foods corresponds to more than half of the calories consumed, and the recent slowdown in the growth of the consumption of ultra-processed foods.

The first and most important of these resistances is the strong food culture based on fresh or minimally processed foods followed by the majority of the Brazilian population on a daily basis, and not only on festive occasions^23^. Public policies and other actions to implement the Brazilian Dietary Guidelines also play a crucial role (Chart 2). In the scope of public policies, the update of the regulations of the National School Feeding Program is the most emblematic example, restricting the supply of ultra-processed foods in schools. Another highly relevant policy is the recent review of the composition of the national basic food basket, which completely excludes ultra-processed foods^26^.

**Chart 2.** Examples of public policies and other measures to implement and disseminate the Dietary Guidelines for the Brazilian Population.

Measure	Description
Updating the composition of the national basic food basket	By means of Decree No. 11,936 of 2024, the composition of the Brazilian basic food basket was updated, completely excluding ultra-processed foods. The new basic food basket expanded and diversified the number of fresh and minimally processed items, seeking to value the regional diversity in the country. The objective of the decree is to guide actions, policies and programs related to the food production, supply and consumption, such as the taxation of foods within the scope of the tax reform sanctioned in 2025.
Regulation of food prices through tax reform	Through Complementary Bill No. 68/2024, which regulates the tax reform on consumption, all items in the new national basic food basket were exempted from taxes, with a view to reducing the prices of foods recommended by the Guidelines. In contrast, sugary drinks were the only category of ultra-processed foods included in the selective tax list, which includes items considered harmful to health or the environment.
Creation of the Solidarity Kitchen Program	By means of Decree No. 11,937 of 2024, the Solidarity Kitchen Program was created, which aims to provide free and quality food to the population, preferably to people in situations of vulnerability and social risk. One of the program’s principles is the promotion of adequate and healthy eating, in accordance with the recommendations of the Guidelines.
Promotion of adequate and healthy food in the school environment	By means of Decree No. 11,821 of 2023, it was established that all actions to promote adequate and healthy eating in the school environment must be referenced in the Guidelines. This includes both food and nutritional education actions as well as the supply, marketing and marketing communication of food and beverages.
Updating the law of the National School Food Program	By means of Resolution No. 6 of 2020, it was established that at least 75% of the transfers from the National Fund for the Development of Education must be allocated to the purchase of fresh or minimally processed foods. The purchase of processed and ultra-processed foods is now limited to 20% of the budget. In 2025, the Federal Government announced that it will reduce this limit to 15% in 2025 and to 10% in 2026, through changes to the 2020 resolution.
Ban on ultra-processed foods in institutional settings of the Ministry of Health	By means of Ordinance No. 1,274 of 2016, the supply, sale and advertising of ultra-processed foods were prohibited in workplaces linked to the Ministry of Health. In line with the ordinance, a booklet was also prepared with guidelines for offering healthy foods in these places.
Ban on ultra-processed foods in school cafeterias	Through subnational legislation, Brazilian municipalities have limited or banned the sale of ultra-processed foods in school cafeterias. Examples are the municipalities of Rio de Janeiro (Municipal Law No. 7,987, of 2023) and Niterói (Municipal Law No. 3,766, of 2023), both in the state of Rio de Janeiro.
Development and dissemination of protocols for dietary advice based on the Dietary Guidelines aimed at primary health care (PHC) professionals of the Unified Health System (SUS)	In partnership with the Center for Epidemiological Research in Nutrition and Health at the University of São Paulo (NUPENS/USP), the Ministry of Health has made available a series of five Protocols for Individual Dietary Advice Based on the Brazilian Dietary Guidelines, to serve as a support tool for PHC professionals. Each booklet focuses on a life cycle: children aged 2 to 10, adolescents, adults, pregnant women and older people. A course aimed at qualifying PHC professionals to use the protocols, QualiGuia, was developed and made available to PHC professionals throughout the country through the Universidade Aberta-SUS platform. (https://www.unasus.gov.br/cursos/curso/47024).
Preparation of health education materials for the general population and support for PHC professionals	Several health education materials based on the Dietary Guidelines have been developed and made available for different audiences, such as folders, videos, booklets and manuals, under agreements between the Ministry of Health and public education and research institutions in Brazil. Some examples are the instructional manuals for individual and collective management of obesity in the SUS (Federal University of Minas Gerais), a series of short videos disseminating messages from the guide (NUPENS/USP) and activity books for promoting adequate and healthy eating based on the guide (State University of Rio de Janeiro). The publications can be accessed at: https://www.gov.br/saude/pt-br/composicao/saps/promocao-da-saude/guias-alimentares/publicacoes

In the Unified Health System (SUS), the Brazilian Dietary Guidelines is recognized as a central theoretical reference for actions to promote healthy eating, particularly in primary care. There are numerous documents that support its use, such as the Protocols based on the Brazilian Dietary Guidelines for Individual Dietary Advice, which guide professionals during their consultations^27^.

Finally, access to sound information by the population is also a significant, albeit underestimated, obstacle. The dissemination of this knowledge occurs on different fronts. At the institutional level, the Ministry of Health itself has produced educational materials for the general public. At the same time, independent communicators with a wide reach, such as Rita Lobo, have played an important role in popularizing the concept of ultra-processed foods. In addition, civil society organizations such as the Brazilian Institute for Consumer Protection (Idec) and the Alliance for Adequate and Healthy Food (ACT Health Promotion), promote campaigns aimed at raising awareness about the impacts of these products on health and the environment.

The mainstream media, in turn, has increasingly given space to the topic. Since the publication of the Dietary Guidelines for the Brazilian Population, there has been a significant increase in reports addressing ultra-processed foods in the country’s main newspapers since 2015, with an acceleration from 2022 onwards^28^, which indicates an increase in the dissemination of the concept among the public. As with the tobacco success story in Brazil, access to information plays a central role in the fight for a social norm that promotes healthy eating.

The new food labeling system, in force in Brazil since 2022, also contributes to this scenario, even though it is not directly based on the Dietary Guidelines or the Nova classification. Front-of-pack nutritional labels that warn of excessive sodium, saturated fat or added sugar content in foods cover a significant portion of ultra-processed foods available for sale in Brazil^29^. Studies conducted in other countries that adopt similar front-of-pack labeling models indicate a reduction in sales of products that receive labels^30^, showing that this type of labeling can be useful in supporting consumers when purchasing food.

## POLITICAL AND SCIENTIFIC CHALLENGES

Important scientific and political challenges still persist. The first concerns the measurement of food consumption according to the Nova classification in epidemiological studies. Despite the relevance of the studies on the subject published to date, a common challenge in applying the Nova classification has been the use of food consumption data collected by non-specific instruments to capture differences in industrial processing. Since they lack this information, they could induce bias and reduce the power to identify significant associations.

A common example of this limitation is the classification of items such as “cake” or “soup”, which, when collected without detailed information on ingredients or preparation method, can be allocated to more than one Nova group. Studies indicate that this categorization can be challenging when performed by untrained evaluators or without the support of standardized protocols. However, research that follows good practices in applying Nova in food databases has shown that traditional questionnaires can achieve satisfactory levels of validity and reliability^31,32,33^.

To overcome this limitation, the NUPENS/USP team developed three new self-administered electronic instruments: Screener-Nova, the Nova 24-h food recall (Nova24h) and the Nova Food Frequency Questionnaire-Nova (NovaFFQ). These instruments have been successfully applied in studies of the NutriNet Brasil cohort. Some examples of studies already conducted include one that showed an association between the caloric participation of ultra-processed foods (estimated by Nova24h) and the risk of depression and another that described the relationship between the scores of ultra-processed foods and fresh or minimally processed foods (estimated by Screener-Nova) and weight gain^34,35^. In addition, the three instruments are available free of charge on the QuestNova online platform (https://questnova.com.br/)^36^.

The second challenge concerns the production of evidence through RCTs. There are few experimental studies that evaluate the effects of ultra-processed foods on the risk of NCDs, although the registration of new protocols suggests that this number should increase soon. The Prevention of Chronic Diseases with the Brazilian Diet (PREDIBRA) study is an ongoing RCT that aims to evaluate the effectiveness of a digital intervention that promotes the Brazilian diet, as proposed by the Brazilian Dietary Guidelines, in preventing health outcomes among participants in the NutriNet Brasil cohort. It will provide evidence of the causal association between the reduction of ultra-processed foods and the prevention of weight gain, in addition to testing a behavior change program aligned with the assumptions of the National Digital Health Strategy, which could be incorporated by SUS.

Finally, one of the most relevant political challenges today is the advancement of the economic regulation of ultra-processed foods. The tax reform represented a partial victory by including soft drinks in the selective tax and exempting items from the basic food basket, which could especially benefit the most vulnerable populations. However, the fight for price regulation of these products continues, since selective taxation could be extended to other ultra-processed foods, in line with the evidence of its benefits, especially for the lower-income population.

Similarly, Brazil still needs to more effectively combat advertising of ultra-processed foods, especially that aimed at children and adolescents. Despite legislation that recognizes as abusive the practice of directing advertising and marketing communications to children and adolescents, its effectiveness is limited by the lack of specific regulations that guarantee the implementation and monitoring of these restrictions.

## CONCLUSION

The creation of the Nova classification and its incorporation into public policies exemplify the transformative impact of nutritional epidemiology in Brazil and around the world. This trajectory has been led by researchers such as Carlos Monteiro, whose leadership was fundamental to the conception and dissemination of Nova, Renata Levy, who contributed significantly to its operationalization in population research, and Patricia Jaime, who played a central role in the incorporation of this evidence into public policies, especially in the formulation of the Dietary Guidelines for the Brazilian Population. These contributions are marked both by continuous conceptual and methodological improvement and by the commitment to transform the health conditions of the population. The trajectory of epidemiology in Brazil has never been restricted to decontextualized quantitative analyses; on the contrary, it has always been based on a radical practice of transdisciplinarity, integrating scientific rationality with the complex health reality of the country and the production of evidence for SUS, under the strong influence of the concepts of social determination of health. Applying this perspective to the study of ultra-processed foods, powerful new insights into the determinants of health emerge, indicating the need for innovative strategies to address them. To combat the ultra-processed food epidemic, it is essential to adopt a holistic approach that goes beyond traditional interventions and recognizes the interconnections between health, society, economy and culture. While the determinants related to the globalized food system are powerful, Brazil offers examples of resilience that can address these challenges. Continued research and policy efforts are crucial to promote healthier diets for the entire population.

## Data Availability

Data sharing is not applicable.

## References

[1] Levy-Costa RB, Sichieri R, Pontes NS, Monteiro CA (2005). Disponibilidade domiciliar de alimentos no Brasil: distribuição e evolução (1974-2003). Rev Saúde Pública.

[2] Monteiro CA (2009). Nutrition and health. The issue is not food, nor nutrients, so much as processing. Public Health Nutr.

[3] Monteiro CA, Levy RB, Claro RM, Castro IRR, Cannon G (2010). A new classification of foods based on the extent and purpose of their processing. Cad Saúde Pública.

[4] Martinez-Steele E, Khandpur N, Batis C, Bes-Rastrollo M, Bonaccio M, Cediel G (2023). Best practices for applying the Nova food classification system. Nat Food.

[5] Louzada ML da C, Martins APB, Canella DS, Baraldi LG, Levy RB, Claro RM (2015). Alimentos ultraprocessados e perfil nutricional da dieta no Brasil. Rev Saúde Pública.

[6] Louzada ML, Baraldi LG, Steele EM, Martins AP, Canella DS, Moubarac JC (2015). Consumption of ultra-processed foods and obesity in Brazilian adolescents and adults. Prev Med.

[7] Louzada ML, Martins AP, Canella DS, Baraldi LG, Levy RB, Claro RM (2015). Impact of ultra-processed foods on micronutrient content in the Brazilian diet. Rev Saúde Pública.

[8] Brasil. Ministério da Saúde (2014). Secretaria de Atenção Básica. Departamento de Atenção Básica. Guia Alimentar Para a População Brasileira.

[9] Louzada ML da C, Canella DS, Jaime PC, Monteiro CA (2019). Alimentação e saúde: a fundamentação científica do Guia Alimentar para a População Brasileira.

[10] Lane MM, Gamage E, Du S, Ashtree DN, McGuinness AJ, Gauci S (2024). Ultra-processed food exposure and adverse health outcomes: umbrella review of epidemiological meta-analyses. BMJ.

[11] Hall KD, Ayuketah A, Brychta R, Cai H, Cassimatis T, Chen KY (2019). Ultra-processed diets cause excess calorie intake and weight gain: an inpatient randomized controlled trial of ad libitum food intake. Cell Metab.

[12] Hamano S, Sawada M, Aihara M, Sakurai Y, Sekine R, Usami S (2024). Ultra-processed foods cause weight gain and increased energy intake associated with reduced chewing frequency: A randomized, open-label, crossover study. Diabetes Obes Metab.

[13] Srour B, Kordahi MC, Bonazzi E, Deschasaux-Tanguy M, Touvier M, Chassaing B (2022). Ultra-processed foods and human health: from epidemiological evidence to mechanistic insights. Lancet Gastroenterol Hepatol.

[14] Juul F, Vaidean G, Parekh N (2021). Ultra-processed foods and cardiovascular diseases: Potential mechanisms of action. Adv Nutr.

[15] Levy RB, Andrade GC, Cruz GLD, Rauber F, Louzada MLDC, Claro RM (2022). Three decades of household food availability according to NOVA Brazil, 1987–2018. Rev Saúde Pública.

[16] Louzada ML, Steele EM, Rezende LFM, Levy RB, Monteiro CA (2022). Changes in obesity prevalence attributable to ultra-processed food consumption in Brazil between 2002 and 2009. Int J Public Health.

[17] Louzada MLC (2024). Epidemiologia nutricional aplicada à obesidade.

[18] Louzada MLC, Cruz GL, Silva KAAN, Grassi AGF, Andrade GC, Rauber F (2023). Consumo de alimentos ultraprocessados no Brasil: distribuição e evolução temporal 2008–2018. Rev Saúde Pública.

[19] Monteiro CA, Cannon G (2019). The role of the transnational ultra-processed food industry in the pandemic of obesity and its associated diseases: Problems and solutions. World Nutr.

[20] Maia EG, Dos Passos CM, Levy RB, Bortoletto Martins AP, Mais LA, Claro RM (2020). What to expect from the price of healthy and unhealthy foods over time? The case from Brazil. Public Health Nutr.

[21] Andrade GC, Caldeira TCM, Mais LA, Bortoletto Martins AP, Claro RM (2024). Food price trends during the COVID-19 pandemic in Brazil. PLoS One.

[22] Machado PP, Claro RM, Canella DS, Sarti FM, Levy RB (2017). Price and convenience: The influence of supermarkets on consumption of ultra-processed foods and beverages in Brazil. Appetite.

[23] Monteiro CA, Cannon G. (2012). The impact of transnational “big food” companies on the South: A view from Brazil. PLoS Med.

[24] Moodie R, Bennett E, Kwong EJL, Santos TM, Pratiwi L, Williams J (2021). Ultra-processed profits: The political economy of countering the global spread of ultra-processed foods A synthesis review on the market and political practices of transnational food corporations and strategic public health responses. Int J Health Policy Manag.

[25] Brasil (2020). Resolução nº 6, de 8 de maio de 2020. Dispõe sobre o atendimento da alimentação escolar aos alunos da educação básica no âmbito do Programa Nacional de Alimentação Escolar - PNAE.

[26] Brasil. Decreto nº 11.936, de 5 de março de (2024). Dispõe sobre a composição da cesta básica de alimentos no âmbito da Política Nacional de Segurança Alimentar e Nutricional e da Política Nacional de Abastecimento Alimentar.

[27] Louzada MLD da C, Tramontt CR, Jesus JGL, Rauber F, Hochberg JRB, Santos TSS (2022). Developing a protocol based on the Brazilian Dietary Guidelines for individual dietary advice in the primary healthcare: Theoretical and methodological bases. Fam Med Community Health.

[28] Jaime PC, Bomfim M (2025). Dez anos do Guia Alimentar para a População Brasileira: história, ciência e política. Epidemiol Serv Saúde.

[29] Borges CA, Khandpur N, Neri D, Duran AC (2022). Comparing Latin American nutrient profile models using data from packaged foods with child-directed marketing within the Brazilian food supply. Front Nutr.

[30] Taillie LS, Reyes M, Colchero MA, Popkin B, Corvalán C (2020). An evaluation of Chile’s Law of Food Labeling and Advertising on sugar-sweetened beverage purchases from 2015 to 2017: A before-and-after study. PLoS Med.

[31] Fangupo LJ, Haszard JJ, Leong C, Heath AM, Fleming EA, Taylor RW (2019). Relative validity and reproducibility of a food frequency questionnaire to assess energy intake from minimally processed and ultra-processed foods in young children. Nutrients.

[32] Oviedo-Solís CI, Monterrubio-Flores EA, Rodríguez-Ramírez S, Cediel G, Denova-Gutiérrez E, Barquera S (2022). A semi-quantitative food frequency questionnaire has relative validity to identify groups of NOVA Food Classification System among Mexican adults. Front Nutr.

[33] Oviedo-Solís CI, Monterrubio-Flores EA, Cediel G, Denova-Gutiérrez E, Barquera S (2022). Relative validity of a semi-quantitative food frequency questionnaire to estimate dietary intake according to the NOVA Classification in Mexican children and adolescents. J Acad Nutr Diet.

[34] Werneck AO, Steele EM, Delpino FM, Lane MM, Marx W, Jacka FN (2024). Adherence to the ultra-processed dietary pattern and risk of depressive outcomes: Findings from the NutriNet Brasil cohort study and an updated systematic review and meta-analysis. Clin Nutr.

[35] Santos FSD, Martinez Steele E, Costa CDS, Gabe KT, Leite MA, Claro RM (2023). Nova diet quality scores and risk of weight gain in the NutriNet-Brasil cohort study. Public Health Nutr.

[36] Louzada ML, Souza TN, Frade E, Gabe K, Patricio G (2024). QuestNova: inovação na avaliação do consumo alimentar segundo o processamento industrial. Rev Saúde Pública.

