# Supplementary material for: Nova food classification system: a contribution from Brazilian epidemiology
Source: Rev Bras Epidemiol. 2025 Jun 2;28:e250027. doi: 10.1590/1980-549720250027 (PMC12129239; doi:10.1590/1980-549720250027)
Supplement: Translation

# Translation: Classificação de alimentos Nova: uma contribuição da epidemiologia brasileira

**DOI:** 10.1590/1980-549720250027.2

**Authors:** Maria Laura da Costa Louzada, Kamila Tiemann Gabe

**Affiliations:** I Universidade de São Paulo, Núcleo de Pesquisas Epidemiológicas em Nutrição e Saúde – São Paulo (SP), Brasil.

**Keywords:** Epidemiologia nutricional, Ingestão de alimentos, Alimento processado, Política nutricional, Guias alimentares

## Abstract

Este ensaio apresenta a classificação de alimentos Nova, uma inovação conceitual da epidemiologia brasileira, descrevendo sua gênese, as evidências científicas derivadas de sua aplicação e suas implicações para a saúde pública. Criada em 2010, a Nova classifica os alimentos em quatro grupos com base no grau de processamento: alimentos *in natura* ou minimamente processados, ingredientes culinários processados, alimentos processados e alimentos ultraprocessados. Desde seu desenvolvimento, diversos estudos epidemiológicos têm demonstrado os impactos negativos do consumo elevado de alimentos ultraprocessados à saúde, como a associação com diversas doenças crônicas não transmissíveis, tais como obesidade, diabetes, doenças cardiovasculares e desfechos de saúde mental. No Brasil, o consumo desses alimentos cresceu significativamente nas últimas décadas, com a participação calórica dos ultraprocessados aumentando de 12,6 para 18,4% entre 2002–2003 e 2017–2018, sendo esse aumento mais intenso entre grupos socioeconômicos mais vulneráveis. A Nova fundamenta as recomendações do Guia Alimentar para a População Brasileira e tem desempenhado papel crucial no embasamento de políticas públicas, como a atualização da cesta básica nacional e as diretrizes do Programa Nacional de Alimentação Escolar, que visam restringir o acesso a alimentos ultraprocessados. Por fim, o ensaio aborda os desafios políticos e científicos, incluindo a necessidade de mais estudos experimentais que fortaleçam as evidências e o potencial de estratégias fiscais e de regulação da publicidade que considerem o impacto do processamento de alimentos na saúde.

## INTRODUÇÃO

No fim da década de 1980, as Pesquisas de Orçamentos Familiares começaram a revelar mudanças notáveis nos padrões de compra de alimentos das famílias brasileiras. De 1987 a 2003, observou-se queda significativa na aquisição de alguns alimentos, como arroz, feijão, farinha, ovos e carne e também de ingredientes culinários, como sal de cozinha, açúcar de mesa e óleos vegetais. Simultaneamente, houve um aumento das compras de bolos industrializados, salgadinhos e bolachas, salsichas, pratos prontos e bebidas adoçadas^1^.

Àquela época, o processamento industrial era praticamente ignorado nos estudos sobre consumo de alimentos, e as conclusões iniciais pareciam levar a um paradoxo: apesar da queda na aquisição de sal, óleos e açúcar — cuja redução era uma recomendação central para combater a obesidade e as doenças crônicas não transmissíveis (DCNT) —, esses problemas de saúde continuaram a aumentar. Investigações motivadas por essa discrepância revelaram que estava em curso uma transição radical nos padrões alimentares da população brasileira.

A queda da aquisição desses alimentos, ao contrário do que seria esperado, não foi acompanhada pela alteração do perfil nutricional da dieta, ou seja, redução do consumo de sódio, açúcar livre e gorduras, mas sim indicava a redução do consumo de alimentos tradicionais e suas preparações culinárias, como arroz e feijão (cujo preparo requer ingredientes culinários), e o concomitante aumento do uso de produtos industrializados.

Com base na observação de que seria necessário um novo paradigma para compreender essa transição, a equipe do Núcleo de Pesquisas Epidemiológicas em Nutrição e Saúde da Universidade de São Paulo (NUPENS/USP) iniciou, em 2009, uma revolucionária linha de pesquisa fundamentada na hipótese de que o processamento industrial era um fator-chave na relação entre alimentação e saúde^2^.

## O SISTEMA DE CLASSIFICAÇÃO DE ALIMENTOS NOVA

Em 2010^3^, foi proposta a classificação de alimentos Nova, que, após atualizações ao longo dos anos, divide todos os alimentos segundo características do processamento industrial em quatro grupos: alimentos *in natura* ou minimamente processados, ingredientes culinários processados, alimentos processados e alimentos ultraprocessados (Quadro 1).

**Quadro 1.** Definição e exemplos dos quatro grupos da classificação de alimentos Nova.

**Grupo 1) Alimentos in natura ou minimamente processados.**
Alimentos in natura são partes comestíveis de plantas (frutas, sementes, folhas, caules, raízes, tubérculos) ou de animais (músculos, vísceras, ovos, leite), além de fungos, algas e água, após separação da natureza. Alimentos minimamente processados são alimentos alterados por processos industriais mínimos, como remoção de partes não comestíveis ou indesejadas, secagem, moagem, trituração, torrefação, fracionamento, tostagem, pasteurização, refrigeração, congelamento, fermentação não alcoólica e outros métodos que não adicionam sal, açúcar, óleos ou gorduras ou outros ingredientes ao alimento original. Esses processos têm como objetivo prolongar a vida útil desses alimentos, permitindo seu armazenamento para uso prolongado e, muitas vezes, facilitar ou diversificar sua preparação. Aditivos geralmente não são necessários e são encontrados apenas excepcionalmente em alimentos minimamente processados. Exemplos: Frutas e vegetais de folhas e raízes frescos, espremidos, refrigerados, congelados ou secos; grãos como arroz integral, parboilizado ou branco, espiga ou grão de milho, grãos de trigo; leguminosas como feijões, lentilhas e grão-de-bico; nozes e sementes oleaginosas cruas ou torradas (sem adição de sal ou açúcar); raízes e tubérculos, como batatas, batata-doce e mandioca; fungos como cogumelos frescos ou secos; ervas e especiarias, como tomilho, orégano, hortelã, pimenta, cravo e canela, inteiros ou em pó, frescos ou secos; sêmolas, flocos ou farinha de milho, trigo, aveia ou mandioca; carne, aves, peixes e frutos do mar, inteiros ou em cortes, frescos, refrigerados ou congelados; ovos; leite fresco ou pasteurizado; iogurte natural fresco ou pasteurizado; sucos de frutas ou vegetais frescos ou pasteurizados (sem adição de açúcar, adoçantes ou aromas); chá, café e água potável. Além disso, alimentos compostos de dois ou mais itens deste grupo, como frutas secas mistas, granola feita de cereais, nozes e frutas secas sem adição de açúcar, mel ou óleo; macarrão, cuscuz e polenta feitos com farinhas, flocos ou sêmolas e água; e alimentos com vitaminas e minerais adicionados, geralmente para repor nutrientes perdidos durante o processamento, como farinha de trigo ou milho fortificada com ferro e ácido fólico.
**Grupo 2) Ingredientes culinários processados.**
Substâncias obtidas diretamente de alimentos do grupo 1 ou da natureza por processos como centrifugação, desidratação, extração, mineração, prensagem e refinação. Esses processos ajudam na criação de produtos usados no tempero e cozimento de alimentos do grupo 1 e seu uso em preparações culinárias. Aditivos geralmente não são necessários e são encontrados apenas excepcionalmente em ingredientes culinários processados. Exemplos: Óleos vegetais extraídos de sementes, nozes ou frutos (notadamente azeitonas); açúcar e melaço obtidos da cana-de-açúcar ou beterraba; mel extraído de favos e xarope de árvores de bordo; vinagre; amidos extraídos do milho e de outras plantas; sal extraído de minas ou da água do mar; manteiga e banha obtidas do leite e da carne de porco. Além disso, produtos compostos de dois itens do grupo 2, como manteiga salgada, e itens do grupo 2 com adição de vitaminas ou minerais, como sal iodado.
**Grupo 3) Alimentos processados.**
Produtos alimentares industrialmente fabricados feitos da adição de ao menos um ingrediente do grupo 2 (como sal, açúcar, óleo ou gordura) a alimentos do grupo 1, por meio de métodos de preservação como enlatamento e engarrafamento e, no caso de pães e queijos, usando fermentação não alcoólica e fervura ou cozimento. Os processos e ingredientes visam aumentar a durabilidade dos alimentos do grupo 1 e modificar ou aprimorar suas qualidades sensoriais. Os alimentos processados frequentemente contêm aditivos que prolongam a duração do produto, protegem propriedades originais ou evitam a proliferação de microrganismos (como conservantes e antioxidantes), mas não aditivos com funções cosméticas (presente em alimentos ultraprocessados). Exemplos: Frutas em calda; vegetais e leguminosas enlatados ou engarrafados em salmoura; nozes e sementes salgadas ou açucaradas; peixes secos ou enlatados. Pães, queijos, pastéis, bolos, biscoitos; carnes curadas; e produtos prontos para aquecer, como hambúrgueres, tortas e pratos de massa e pizza pré-preparados, quando esses produtos são feitos exclusivamente de alimentos do grupo 1 e sal, óleo, açúcar ou outros ingredientes do grupo 2 Nova e não contêm classes de aditivos com função cosmética.
**Grupo 4) Alimentos ultraprocessados.**
Produtos alimentares industrialmente fabricados compostos de vários ingredientes (formulações), incluindo óleos, gorduras, açúcar e sal (geralmente em combinação e em quantidades maiores do que em alimentos processados) e substâncias alimentares de uso culinário raro ou inexistente (como óleos hidrogenados, amidos modificados, xarope de milho com alto teor de frutose e isolados de proteínas). Alimentos do grupo 1 estão ausentes ou representam uma pequena proporção dos ingredientes na formulação. Os processos que permitem a fabricação de alimentos ultraprocessados incluem técnicas industriais como extrusão, moldagem e pré-fritura; aplicação de aditivos, incluindo aqueles cuja função é tornar o produto palatável ou hiperpalatável, como corantes, adoçantes não açucarados, aromatizantes e emulsificantes; e embalagens sofisticadas, geralmente com materiais sintéticos. Os processos e ingredientes aqui são projetados para criar alternativas altamente lucrativas (ingredientes de baixo custo, longa vida útil, marca enfática) e convenientes (prontos para comer ou beber) a todos os outros grupos alimentares da Nova. Os alimentos ultraprocessados são operacionalmente distinguíveis dos alimentos processados pela presença de substâncias alimentares de uso culinário raro ou inexistente (variedades de açúcares, como xarope de milho com alto teor de frutose, frutose, açúcar invertido, concentrados de suco de frutas, dextrose, maltodextrina e lactose; amidos modificados; óleos modificados, como óleos hidrogenados ou interesterificados; e fontes de proteínas, como glúten, isolado de proteína de soja, caseína, soro de leite, proteínas hidrolisadas e carne mecanicamente separada) ou de aditivos com funções cosméticas (realçadores de sabor, aromas, corantes, emulsificantes, adoçantes, sais emulsificantes, espessantes e agentes de volume, carbonatação, espumação, gelificação, glacê, antiespumantes). Exemplos: Sucos de frutas reconstituídos e bebidas de frutas; refrigerantes carbonatados; bebidas lácteas e bebidas energéticas; iogurte com sabor; guloseimas; margarinas; salsichas, cachorros-quentes, nuggets de frango e peixe, produtos de carne reconstituída; substitutos de carne à base de plantas; cereais matinais extrusados; sopas instantâneas, macarrão e sobremesas em pó; fórmulas infantis e leites de transição; produtos de saúde e emagrecimento, como shakes e pós substitutos de refeições. Pães, pastéis, bolos, biscoitos; carnes curadas; lanches doces ou salgados; e produtos prontos para aquecer, como hambúrgueres, tortas, pratos de massa e pizza pré-preparados, quando esses produtos são compostos de substâncias alimentares de uso culinário raro ou inexistente e/ou contêm classes de aditivos com função cosmética.

Fonte: Adaptado de Martinez-Steele et al.^4^.

Os alimentos ultraprocessados são definidos como formulações industriais prontas para consumo, elaboradas com diversas etapas de processamento industrial e partes de componentes alimentares (como gorduras, açúcares, amido, proteínas isoladas), com pouca ou nenhuma presença de matriz alimentar original e muitas vezes adicionadas de muitos aditivos cosméticos que os tornam mais atraentes. Uma forma prática de identificar esses alimentos é pela análise da lista de ingredientes, buscando um dos seguintes marcadores:

- aditivos alimentares cosméticos, *i.e.*, que modificam as características sensoriais do produto, como aromatizante, corante, edulcorante, espessante, realçador de sabor;
- substâncias de raro uso culinário, como frutose, xarope de milho com alto teor de frutose, concentrados de suco de frutas, açúcar invertido, maltodextrina, dextrose, lactose, óleos hidrogenados, isolado de proteína de soja, caseína e carne mecanicamente separada.

O artigo “Best practices for applying the Nova food classification system”^4^ propõe práticas recomendadas para aplicar a classificação Nova, visando melhorar a precisão da categorização, especialmente quando faltam informações detalhadas sobre a origem, forma de preparo e ingredientes presentes nos alimentos.

## ALIMENTOS ULTRAPROCESSADOS E SAÚDE: EVIDÊNCIAS CIENTÍFICAS

As primeiras evidências do impacto negativo da substituição de alimentos *in natura* ou minimamente processados pelos alimentos ultraprocessados na saúde foram produzidas com base em dados de inquéritos brasileiros de consumo alimentar individual. Os estudos mostraram associações transversais entre a alta participação de alimentos ultraprocessados na alimentação, a deterioração da qualidade nutricional da dieta (tanto em relação aos macronutrientes quanto aos micronutrientes) e a maior chance de ocorrência de obesidade^5,6,7^. Esses artigos foram a base das evidências do Guia Alimentar para a População Brasileira, publicado pelo Ministério da Saúde em 2014^8,9^, tendo como recomendação central a redução do consumo de alimentos ultraprocessados e o fortalecimento do padrão alimentar tradicional brasileiro, baseado em alimentos *in natura* ou minimamente processados.

Com isso, o sistema Nova passou a ser aplicado em dados de diversos estudos de coorte no mundo todo, muitos com amostras grandes e de longa duração, que começaram a ser reanalisados à luz da nova classificação. Diversos estudos foram publicados por grupos de pesquisa independentes mostrando o impacto negativo dos alimentos ultraprocessados na saúde. Recentemente, uma revisão guarda-chuva sumarizou esses estudos e demonstrou que o alto consumo de alimentos ultraprocessados se associou a maior risco de mais de 30 desfechos negativos em saúde, incluindo diabetes, doenças cardiovasculares e até mesmo depressão^10^. Apesar das limitações dos estudos observacionais, como o potencial viés de confusão residual e erros na mensuração, a associação com maior risco para diversas DCNT foi consistentemente observada em vários grandes estudos de coorte, a maioria de alta qualidade, reforçando a plausibilidade de uma relação causal.

Evidências complementares vêm de ensaios clínicos randomizados (ECR). Hall et al.^11^ compararam os efeitos da oferta de uma dieta ultraprocessada (~80% de alimentos ultraprocessados) e de uma dieta não processada (sem alimentos ultraprocessados). Os participantes podiam comer livremente, mas os cardápios oferecidos eram os mesmos em termos de calorias, carboidratos, açúcar, gordura, sódio e fibras. Seus resultados mostraram que, quando expostos a dietas ultraprocessadas, os participantes consumiram, em média, 508 kcal a mais por dia do que quando expostos a dietas sem alimentos ultraprocessados e que, após duas semanas, eles ganharam 0,9 kg de peso consumindo a dieta ultraprocessada e reduziram a mesma quantidade de peso consumindo a dieta não ultraprocessada^11^. Resultados semelhantes foram observados em um ECR japonês no qual participantes que seguiram uma dieta ultraprocessada por uma semana ganharam, em média, 2,2 kg de peso corporal e 0,7 kg de massa gorda. Em contraste, aqueles que consumiram a dieta não ultraprocessada ganharam 1,1 kg de peso corporal, mas reduziram 0,4 kg de massa gorda^12^.

Ademais, vários estudos começaram a levantar hipóteses de que os mecanismos que ligam os alimentos ultraprocessados a doenças vão muito além do perfil de nutrientes. Estudos mostram que os ultraprocessados, por seu apelo de conveniência e hiperpalatabilidade, substituem os alimentos *in natura* ou minimamente processados reconhecidamente saudáveis, tais como leguminosas, legumes, verduras e frutas. A destruição dos elementos naturais da matriz alimentar pelas técnicas de processamento compromete o consumo de compostos bioativos relevantes para a saúde cardiovascular. Os alimentos ultraprocessados aumentam o contato com xenobióticos tóxicos, como acrilamida e acroleína (neoformados em processamentos com uso de carboidratos) e bisfenol A (liberado de embalagens plásticas), e a ingestão de aditivos alimentares, como corantes e aromatizantes. Estes últimos, por mais que sejam regulados por lei, têm cada vez mais se mostrado prejudiciais, particularmente a longo prazo. Por fim, os alimentos ultraprocessados possuem baixo teor de saciedade, podem induzir comportamentos relacionados à adicção e, dessa forma, levam ao consumo excessivo de calorias^13,14^.

## TENDÊNCIA TEMPORAL DO CONSUMO DE ALIMENTOS ULTRAPROCESSADOS NO BRASIL

No Brasil, observa-se a substituição dos alimentos *in natura* ou minimamente processados e de ingredientes culinários por alimentos ultraprocessados. Um estudo utilizando dados de aquisição de alimentos da Pesquisa de Orçamentos Familiares revelou um crescimento da participação desses alimentos no total de calorias adquiridas no domicílio de 12,6 para 18,4% entre 2002–2003 e 2017–2018. Nas regiões metropolitanas, essa tendência é constatada desde a década de 1980, com aumento de 10 para 24% do total de energia adquirida nos domicílios entre 1987–1988 e 2017–2018. O maior crescimento da participação de alimentos ultraprocessados foi verificado entre 1995 e 2003, seguido por uma desaceleração recente, atribuída principalmente à redução das compras de refrigerantes entre 2009 e 2018^15^.

Em outro estudo, quantificou-se a fração do aumento da prevalência da obesidade atribuível ao aumento do consumo de ultraprocessados no Brasil entre 2002 e 2009. A comparação de dois inquéritos nacionais, com dados da aquisição domiciliar de alimentos ultraprocessados e de obesidade, juntamente com dados da estimativa de risco associado ao consumo desses alimentos, demonstrou que o aumento do consumo de alimentos ultraprocessados no intervalo de sete anos foi responsável por mais de um quarto (28,6%) do aumento da prevalência de obesidade no mesmo período^16^.

Desde 2008, também há um inquérito nacional que avalia o consumo individual dos participantes dentro e fora de casa. Esses dados revelaram que, entre 2009 e 2017, houve tendência de aumento da participação de alimentos ultraprocessados na dieta, chegando a quase 20% das calorias consumidas e variando de forma importante entre estratos sociodemográficos (Figura 1). Por exemplo, pessoas brancas apresentaram maior consumo de alimentos ultraprocessados ao longo de todo o período, no entanto o consumo de alimentos ultraprocessados aumentou significativamente entre as pessoas de raça/cor negra e indígena, mas não entre as pessoas brancas. Em relação à área de moradia, os moradores das regiões urbanas têm consumo mais elevado, e, embora o aumento tenha sido observado tanto no estrato urbano quanto no rural, ele foi mais intenso entre os moradores da região rural^18^.

**Figura 1.** Tendência temporal do consumo de alimentos ultraprocessados (% do total de energia) no Brasil segundo características sociodemográficas e econômicas. Pesquisas de Orçamentos Familiares 2008–2009 e 2017–2018.

Fonte: Adaptada de Louzada^17^.

Viu-se também que, no Brasil, ainda há relação direta entre renda e consumo de alimentos ultraprocessados, sendo o consumo destes maior entre pessoas mais ricas. Todavia, o consumo de ultraprocessados aumentou significativamente nos três grupos de mais baixa renda familiar, estabilizou-se no grupo de renda média alta e diminuiu significativamente no grupo de renda mais alta^18^. Portanto, ao longo desses 10 anos, verificou-se uma padronização do consumo nacional em um nível mais elevado, prejudicando ainda mais os estratos socioeconômicos mais vulnerabilizados.

## DETERMINANTES DO CONSUMO DE ALIMENTOS ULTRAPROCESSADOS

No Brasil, a ascensão do consumo de alimentos ultraprocessados é um fenômeno relativamente recente, que remonta à década de 1980, quando as políticas econômicas neoliberais abriram o Sul Global para a entrada das transnacionais de alimentos ultraprocessados. A globalização do sistema alimentar começou a transformar ambientes locais^19^.

Entre os principais impulsionadores do aumento do consumo de alimentos ultraprocessados, está o aumento do acesso físico e do acesso financeiro a esses alimentos. Em 1995, os alimentos ultraprocessados constituíam o grupo de alimentos mais caro. Desde o início dos anos 2000, o preço desses alimentos sofreu sucessivas reduções, diminuindo a distância que os separa dos alimentos *in natura* ou minimamente processados^20^. Dados mais atuais mostram que a crise resultante da pandemia de COVID-19 acelerou esse processo, e essa inversão de preços já está acontecendo^21^.

Os supermercados têm substituído progressivamente os locais de compras mais tradicionais, como feiras e sacolões, oferecendo uma grande variedade de alimentos ultraprocessados a preços mais baixos do que os observados em outros locais^22^. Além disso, a publicidade dirigida às comunidades de baixo rendimento também ajudou a acelerar a penetração desses produtos nos estratos socioeconômicos mais baixos da população. Dois exemplos são a venda porta a porta de alimentos ultraprocessados em comunidades periféricas e o navio da Nestlé vendendo seus produtos em comunidades amazônicas^23^.

Outro fator crucial que favorece o maior consumo dos alimentos ultraprocessados é a atuação política das grandes corporações do setor e os conflitos de interesse. Indústrias transnacionais de ultraprocessados dispõem de vastos recursos para desenvolver atividades políticas corporativas voltadas à defesa de seus interesses, o que impacta diretamente a formulação e implementação de políticas de saúde pública. Essas estratégias incluem *lobby* direto com tomadores de decisão, uso de instrumentos jurídicos para bloquear regulações, campanhas de comunicação voltadas a influenciar a opinião pública e construção de narrativas que destacam o papel do setor na economia. Além disso, são comuns as ações para estreitar laços com profissionais de saúde e pesquisadores, buscando conferir credibilidade aos posicionamentos da indústria^24^.

Em contrapartida, há as forças contrárias. Elas ajudam a explicar o fato de a magnitude de consumo observada no Brasil ser inferior à verificada em outros países, como os Estados Unidos, em que o consumo desses alimentos corresponde a mais da metade das calorias consumidas, e a desaceleração recente do crescimento do consumo de alimentos ultraprocessados.

A primeira e mais importante dessas resistências é a cultura alimentar forte baseada em alimentos *in natura* ou minimamente processados seguida pela maior parte da população brasileira no dia a dia, e não somente em ocasiões festivas^23^. As políticas públicas e outras ações de implementação do guia brasileiro também desempenham papel crucial (Quadro 2). No âmbito das políticas públicas, a atualização das normas do Programa Nacional de Alimentação Escolar^25^ é o exemplo mais emblemático, restringindo a oferta de alimentos ultraprocessados nas escolas. Outra política de grande relevância é a recente revisão da composição da cesta básica nacional, que exclui completamente os ultraprocessados^26^.

**Quadro 2.** Exemplos de políticas públicas e outras ações de implementação e disseminação do Guia Alimentar para a População Brasileira.

Ação	Descrição
Atualização da composição da cesta básica nacional	Por meio do Decreto nº 11.936, de 2024, foi atualizada a composição da cesta básica brasileira, excluindo completamente os alimentos ultraprocessados. A nova cesta básica ampliou e diversificou o número de itens in natura e minimamente processados, buscando valorizar a diversidade regional no país. O objetivo do decreto é orientar ações, políticas e programas relacionados à produção, ao abastecimento e ao consumo de alimentos, a exemplo da tributação de alimentos no âmbito da reforma tributária sancionada em 2025.
Regulação de preço dos alimentos por meio da reforma tributária	Por meio do Projeto de Lei Complementar nº 68/2024, que regulamentou a reforma tributária sobre o consumo, todos os itens da nova cesta básica nacional foram desonerados de impostos, com vistas a reduzir os preços de alimentos recomendados pelo guia alimentar. Em contrapartida, as bebidas açucaradas foram a única categoria de ultraprocessados incluída na lista do imposto seletivo, que incide sobre itens considerados prejudiciais à saúde ou ao meio ambiente.
Criação do Programa Cozinha Solidária	Por meio do Decreto nº 11.937, de 2024, foi criado o Programa Cozinha Solidária, que tem como objetivo fornecer alimentação gratuita e de qualidade à população, preferencialmente às pessoas em situação de vulnerabilidade e risco social. O programa tem como um de seus princípios a promoção da alimentação adequada e saudável, em acordo às recomendações do guia alimentar.
Promoção da alimentação adequada e saudável no ambiente escolar	Por meio do Decreto nº 11.821, de 2023, foi estabelecido que toda ação de promoção da alimentação adequada e saudável no ambiente escolar deve ser referenciada no guia alimentar. Isso inclui tanto ações de educação alimentar e nutricional quanto de fornecimento, comercialização e comunicação mercadológica de alimentos e bebidas.
Atualização da lei do Programa Nacional de Alimentação Escolar	Por meio da Resolução nº 6, de 2020, foi estabelecido que no mínimo 75% dos repasses do Fundo Nacional de Desenvolvimento da Educação devem ser destinados à compra de alimentos in natura ou minimamente processados. A aquisição de alimentos processados e ultraprocessados passou a ser limitada a 20% do orçamento. Em 2025, o Governo Federal anunciou que reduzirá esse limite para 15% ainda em 2025 e para 10% em 2026, por meio de alterações na resolução de 2020.
Proibição de alimentos ultraprocessados nos ambientes institucionais do Ministério da Saúde	Por meio da Portaria nº 1.274, de 2016, foram proibidos o fornecimento, a comercialização e a publicidade de alimentos ultraprocessados em ambientes de trabalho vinculados ao Ministério da Saúde. Alinhado à portaria, foi também elaborada uma cartilha com orientações para oferta de alimentos saudáveis nesses locais.
Proibição de alimentos ultraprocessados em cantinas escolares	Por meio de legislações subnacionais, municípios brasileiros têm limitado ou banido a comercialização de alimentos ultraprocessados em cantinas escolares. Exemplos são os municípios do Rio de Janeiro (Lei Municipal nº 7.987, de 2023) e Niterói (Lei Municipal nº 3.766, de 2023), ambos no estado do Rio de Janeiro.
Elaboração e disseminação de protocolos para orientação alimentar baseados no guia alimentar voltados para profissionais da atenção primária à saúde (APS) do Sistema Único de Saúde (SUS)	Em parceria com o Núcleo de Pesquisas Epidemiológicas em Nutrição e Saúde da Universidade de São Paulo (NUPENS/USP), o Ministério da Saúde disponibilizou uma série de cinco protocolos de uso do Guia Alimentar para a População Brasileira na Orientação Alimentar, para servir como instrumento de apoio a profissionais da APS. Cada fascículo é voltado a um ciclo da vida: crianças de 2 a 10 anos, adolescentes, adultos, gestantes e idosos. Um curso voltado para a qualificação de profissionais da APS para uso dos protocolos, o QualiGuia, foi desenvolvido e disponibilizado a profissionais da APS de todo o país por meio da plataforma Universidade Aberta-SUS (https://www.unasus.gov.br/cursos/curso/47024).
Elaboração de materiais de educação em saúde para a população em geral e de apoio a profissionais da APS	Foram desenvolvidos e disponibilizados diversos materiais de educação em saúde baseados no guia alimentar voltados para diferentes públicos, como fôlderes, vídeos, cartilhas e manuais, no âmbito de convênios entre o Ministério da Saúde e instituições públicas de ensino e pesquisa no Brasil. Alguns exemplos são os manuais instrutivos para manejo individual e coletivo da obesidade no SUS (Universidade Federal de Minas Gerais), uma série de vídeos curtos de disseminação de mensagens do guia (NUPENS/USP) e os cadernos de atividades de promoção da alimentação adequada e saudável baseados no guia (Universidade do Estado do Rio de Janeiro). As publicações podem ser acessadas em: https://www.gov.br/saude/pt-br/composicao/saps/promocao-da-saude/guias-alimentares/publicacoes

No Sistema Único de Saúde (SUS), o guia brasileiro é reconhecido como referencial teórico central para ações de promoção da alimentação saudável, particularmente na atenção básica. Existem inúmeros documentos que apoiam seu uso, como os Protocolos de Uso do Guia Alimentar, que orientam os profissionais durante suas consultas^27^.

Por fim, o acesso à informação idônea pela população também é uma resistência significativa, embora subestimada. A disseminação desse conhecimento ocorre por diferentes frentes. No âmbito institucional, o próprio Ministério da Saúde tem produzido materiais educativos ao público em geral. Paralelamente, comunicadores independentes de grande alcance, como Rita Lobo, têm desempenhado papel relevante na popularização do conceito de alimentos ultraprocessados. Além disso, organizações da sociedade civil, como o Instituto Brasileiro de Defesa do Consumidor e a Aliança pela Alimentação Adequada e Saudável (ACT Promoção da Saúde), promovem campanhas voltadas à conscientização sobre os impactos desses produtos na saúde e no meio ambiente.

A grande mídia tradicional, por sua vez, tem dado cada vez mais espaço à temática. Desde a publicação do Guia Alimentar para a População Brasileira, observou-se um crescimento significativo das reportagens abordando os alimentos ultraprocessados nos principais jornais do país desde 2015, com uma aceleração de 2022 em diante^28^, o que sinaliza um aumento da disseminação do conceito entre a população. A exemplo do que aconteceu com a história de sucesso do tabaco no Brasil, o acesso à informação tem papel central na disputa de uma norma social pró-alimentação saudável.

A nova rotulagem de alimentos, vigente no Brasil desde 2022, também contribui com esse cenário, ainda que não seja diretamente pautada no guia ou na classificação Nova. Os selos de rotulagem nutricional frontal que alertam para o conteúdo excessivo de sódio, gordura saturada ou açúcar adicionado nos alimentos cobrem uma parcela importante dos alimentos ultraprocessados disponíveis para venda no Brasil^29^. Estudos realizados em outros países que adotam modelos parecidos de rotulagem frontal apontam para a redução das vendas de produtos que recebem selos^30^, mostrando que esse tipo de rotulagem pode ser útil para apoiar os consumidores na hora da compra de alimentos.

## DESAFIOS POLÍTICOS E CIENTÍFICOS

Ainda persistem desafios científicos e políticos importantes. O primeiro refere-se à mensuração do consumo de alimentos segundo a classificação Nova nos estudos epidemiológicos. Apesar da relevância dos estudos sobre o tema publicados até hoje, um desafio comum da aplicação da classificação Nova foi o uso de dados de consumo alimentar coletados por instrumentos inespecíficos para captarem diferenças de processamento industrial. Por não terem essas informações, eles poderiam induzir a vieses e reduzir o poder da identificação de associações significativas.

Um exemplo comum dessa limitação é a classificação de itens como “bolo” ou “sopa”, que, quando coletados sem informações detalhadas sobre ingredientes ou forma de preparo, podem ser alocados em mais de um grupo da Nova. Estudos indicam que essa categorização pode ser desafiadora quando realizada por avaliadores não treinados ou sem o suporte de protocolos padronizados, no entanto pesquisas que seguem boas práticas na aplicação da Nova em bancos de dados alimentares demonstraram que questionários tradicionais podem alcançar níveis satisfatórios de validade e confiabilidade^31,32,33^.

Para superar essa limitação, a equipe do NUPENS/USP desenvolveu três novos instrumentos eletrônicos autoaplicáveis: o Screener-Nova, o recordatório alimentar de 24h-Nova (R24h-Nova) e o Questionário de Frequência Alimentar-Nova (QFA-Nova). Esses instrumentos vêm sendo aplicados com sucesso em estudos da coorte NutriNet-Brasil. Alguns exemplos de estudos já realizados incluem um que mostrou associação entre a participação calórica de ultraprocessados (estimada pelo R24h-Nova) e o risco de depressão e outro que descreveu a relação entre os escores de alimentos ultraprocessados e de alimentos *in natura* ou minimamente processados (estimados pelo Screener-Nova) e o ganho de peso^34,35^. Além disso, os três instrumentos estão disponíveis gratuitamente na plataforma *online* QuestNova (https://questnova.com.br/)^36^.

O segundo desafio refere-se à produção de evidências por meio de ECR. São escassos os estudos de delineamento experimental que avaliem os efeitos dos alimentos ultraprocessados no risco de DCNT, embora o registro de novos protocolos sugira que esse número deva crescer em breve. O estudo Prevenção de Doenças Crônicas com a Dieta Brasileira (PREDIBRA) é um ECR em andamento que tem como objetivo avaliar a eficácia de uma intervenção digital que promove a dieta brasileira, conforme proposta pelo guia brasileiro, na prevenção de desfechos de saúde entre participantes da coorte NutriNet Brasil. Ele fornecerá evidências da associação causal entre a redução de alimentos ultraprocessados e a prevenção de ganho de peso, além de testar um programa de mudança de comportamento alinhado aos pressupostos da Estratégia Nacional de Saúde Digital, que poderá ser incorporado pelo SUS.

Por fim, um dos desafios políticos mais relevantes atualmente é o avanço na regulação econômica dos alimentos ultraprocessados. A reforma tributária representou uma vitória parcial ao incluir os refrigerantes no imposto seletivo e desonerar os alimentos da cesta básica, o que pode beneficiar especialmente as populações mais vulneráveis, no entanto a luta pela regulação de preços desses produtos continua, uma vez que a tributação seletiva poderia ser ampliada para outros ultraprocessados, alinhando-se às evidências dos seus benefícios, especialmente para a população de menor renda.

De forma semelhante, o Brasil ainda carece de um enfrentamento mais efetivo da publicidade de alimentos ultraprocessados, especialmente aquela voltada para crianças e adolescentes. Apesar de legislações que reconhecem como abusiva a prática de direcionamento de publicidade e comunicação mercadológica ao público infantojuvenil, sua efetividade é limitada pela ausência de regulamentações específicas que garantam a implementação e fiscalização dessas restrições.

## CONCLUSÃO

A criação da classificação Nova e sua incorporação em políticas públicas exemplificam o impacto transformador da epidemiologia nutricional no Brasil e no mundo. Essa trajetória tem sido protagonizada por pesquisadores como Carlos Monteiro, cuja liderança foi fundamental para a concepção e disseminação da Nova, Renata Levy, que contribuiu significativamente para sua operacionalização em pesquisas populacionais, e Patrícia Jaime, que desempenhou papel central na incorporação dessas evidências nas políticas públicas, especialmente na formulação do Guia Alimentar para a População Brasileira. Essas contribuições são marcadas tanto pelo contínuo aprimoramento conceitual e metodológico quanto pelo compromisso de transformar as condições de saúde da população. A trajetória da epidemiologia no Brasil nunca se restringiu a análises quantitativas descontextualizadas; pelo contrário, sempre esteve fundamentada em uma prática radical de transdisciplinaridade, integrando a racionalidade científica com a complexa realidade sanitária do país e a produção de evidências para o SUS, sob a forte influência das concepções de determinação social da saúde.

Aplicando essa perspectiva ao estudo dos alimentos ultraprocessados, novas e poderosas compreensões sobre os determinantes de saúde emergem, indicando a necessidade de estratégias inovadoras para enfrentá-los. Para combater a epidemia dos alimentos ultraprocessados, é essencial adotar uma abordagem holística que vá além das intervenções tradicionais e reconheça as interconexões entre saúde, sociedade, economia e cultura. Embora os determinantes relacionados ao sistema alimentar globalizado sejam poderosos, o Brasil oferece exemplos de resistências que podem enfrentar esses desafios. Continuar a pesquisa e os esforços políticos é crucial para promover uma alimentação mais saudável para toda a população.

## Data Availability

O compartilhamento de dados não é aplicável
